# The Relationship between the Morphology and Structure and the Quality of Fruits of Two Pear Cultivars (*Pyrus communis* L.) during Their Development and Maturation

**DOI:** 10.1155/2013/846796

**Published:** 2013-11-13

**Authors:** Agata Konarska

**Affiliations:** Department of Botany, Faculty of Horticulture and Landscape Architecture, University of Life Sciences in Lublin, Akademicka 15, 20-950 Lublin, Poland

## Abstract

The flavour and nutritional values of pears are appreciated by consumers worldwide, who, however, demand specific fruit quality, that is, attractive appearance, firmness and flavour, and health safety as well as long-term shelf life and storability. Pear cultivars differ in terms of the above-mentioned traits; therefore, we undertook investigations to demonstrate the differences in structure of fruits of two pear cultivars that determine fruit quality in its broadest sense. The micromorphology, anatomy, and ultrastructure of “Clapp's Favourite” and “Conference” fruits in the fruit set stage and in the harvest maturity stage were investigated under light microscope and scanning and transmission electron microscopes. The fruits of “Clapp's Favourite” and “Conference” in the fruit set stage exhibited distinct differences in the values of anatomical parameters only. Substantial differences in fruit structure were observed in the harvest maturity stage. The analyses indicate that firmness and durability of pear fruits are largely influenced by the presence of russeting, the proportion of closed lenticels and number of stone cells, and the content of starch grains and tannin compounds. The thickness of the cuticle and presence of epicuticular waxes as well as the number of lenticels and the number and depth of microcracks play a minor role.

## 1. Introduction

Pears are one of the oldest plants cultivated by man. For several years, there has been increased interest in consumption of pears; hence, areas of cultivation thereof have been increasing, production has been intensified, and new cultivars have been developed. The great demand for the fruit is associated with their taste and nutritional values. Pears are characterised by attractive flavour, aroma, and juiciness as well as high contents of potassium, fibre, vitamin C, and iodine. Compared with apples, pears cause allergic reactions less frequently. They are low in calories, stimulate digestion and bowel peristaltic movements, have diuretic, antipyretic, and antitussive activity, and regulate blood pressure [1, 2].

Pear trees have higher climatic and soil requirements than apple trees and their fruit is more difficult to store [3, 4]. Moreover, pears transpire more intensively than apples and are more susceptible to mechanical damage [5]. Pear storability is associated with the cultivar, fruit harvest maturity, and storage conditions [6–8]. Only a few among the numerous pear cultivars have satisfactory appearance and flavour and long-term storability. Summer cultivars can be stored over a relatively short period (a few weeks), while the storage time of winter cultivars extends over several months, particularly in controlled-atmosphere conditions [9, 10]. Fruit quality is influenced by external conditions, for example, temperature, humidity, and fruit health, and by internal factors related to the fruit structure. A particularly important function is ascribed to the structure of the fruit surface layer and the structure of parenchymal cells [11, 12].

Among more than 5000 pear cultivars, “Clapp's Favourite” and “Conference” are highly valued in amateur cultivation and commercial orchards in Europe [2]. “Clapp's Favourite” is an early-autumn cultivar, whose fruits ripen in late July and early August. They should be consumed directly after harvest (between the first decade and late August) or they can be stored over a short period (7–10 weeks in a standard cold-storage facility). “Conference” is a late-autumn cultivar; its fruits reach harvest maturity in late September and early October and consumption maturity after a period of storage under low temperature, which may last 5-6 months depending on the type of the cold-storage facility. “Clapp's Favourite” fruits are characterised by a relatively thick, glossy, and greasy peel, whereas the peel in the “Conference” is dry, matt, and usually with partial russeting [1, 3, 9].

Given the huge popularity of “Clapp's Favourite” and “Conference” pears among consumers as well as their different surface and storability, the aim of the present study is to demonstrate differences in developing and ripening “Clapp's Favourite” and “Conference” fruits that exert an effect on fruit firmness, length of shelf life, and storability. The investigations were conducted using light microscopy and scanning and transmission electron microscopy.

## 2. Materials and Methods

Fruits of two pear cultivars, Clapp's Favourite and Conference, were examined in the years 2011-2012 in two periods: stage I: fruit set stage with a diameter of 1 cm harvested 21 days after anthesis (May 15–20) and stage II: harvested at the preclimacteric stage. For “Clapp's Favourite” it was late September and for “Conference” it was late October. The fruits came from a private orchard in the Lublin region (Poland) in which conventional growing methods were used. Twenty medium-sized, similarly coloured fruits that were free of defect were collected from the central part of randomly chosen trees. Special care was taken to avoid touching the fruit surface area intended for observation while picking, transporting, and preparing the pear to SEM (to avoid rubbing off and destruction of the wax layer). Further investigations were carried out using fragments of fruit and peel sampled from the equatorial part of the fruit.

### 2.1. Scanning Electron Microscopy (SEM)

Typical fixation of the material for SEM investigations involves dehydration, which can remove or alter lipids that form the wax coating on the apple surface, and critical point drying can shrink and destroy tissues [13]. Therefore, a modified and simplified methodology was used in order to prevent destruction of the epicuticular wax. Fragments of fruit with peel (5 mm × 5 mm × 1 mm) were sampled from each cultivar immediately after the fruits had been collected from the trees. In the case of the “Conference,” samples were taken from peel-covered fruit parts and from russeted parts. The samples (freshly cut sections) were gently wiped with a paper towel, carefully mounted onto stubs, sputter-coated with gold, and examined under a TESCAN/VEGA LMU scanning electron microscope at an accelerating voltage of 30 kV. Additionally, in the fruit set stage and in the harvest maturity stage stomata and lenticels in the equatorial fruit parts were counted within an area of 1 cm^2^ of the epidermis in each of the ten “Clapp's Favourite” and “Conference” fruits using the Morphology program coupled with SEM. In “Conference,” the lenticels were counted on the nonrusseted surface only.

### 2.2. Light Microscopy (LM)

Hand-cut cross-sections from fragments of 10 fruits of the “Clapp's Favourite” and “Conference” cultivars were made; next, the samples were embedded in glycerol gelatin and viewed under the Nicon SE 102 light microscopy. In each slide, the thickness of the cuticle, the height of the epidermal cells, the number of layers of hypodermis and its overall thickness, and the thickness of 3 layers of the parenchyma located under the hypodermis were determined in five places. Additionally, the diameter and thickness of the walls of 10 largest stone cells located in the parenchyma adjacent to the hypodermis were measured. Further, the samples of fresh material were stained with Lugol's iodine in order to detect starch grains in leucoplasts, with Sudan III (a saturated alcoholic solution of Sudan III) to detect lipophilic substances in the cuticle and lenticels, and with FeCl_3_ to detect tannin substances. Hand-cut samples obtained from fresh material were also viewed under a stereoscopic Nikon Eclipse 90i microscope to detect the distribution of the cuticle, chlorophyll, and lignified stone cell walls. Photomicrographs were captured using a digital camera (Nikon Fi1) and NIS-Elements Br 2 software, or a Zeiss Axio Imager Z1 fluorescence microscope equipped with an AxioCam MR digital camera.

Semithin transverse sections (0.7 *μ*m thick) were stained with 1% methylene blue with 1% azur II in a 1% aqueous solution of sodium tetraborate. The material was fixed and embedded in synthetic resin with the standard method used in transmission electron microscope. Sections were observed by means of a Nikon Eclipse 90i microscope.

### 2.3. Transmission Electron Microscopy (TEM)

Small samples (2 mm × 2 mm × 2 mm) of “Clapp's Favourite” and “Conference” fruits were fixed in 2% paraformaldehyde and 2.5% glutaraldehyde buffered at pH 7.4 in 0.1 M cacodylate buffer at the fruit set stage and after harvest. Fixation was performed at room temperature for two hours, followed by 12 hr at 4°C. When fixed, the samples were rinsed with 0.1 M cacodylate buffer at 4°C for 24 hr and then treated with 1% OsO_4_. After passage through increasing concentrations of propylene oxide in ethanol and finally through pure propylene oxide, the samples were embedded for 12 hr in Spurr Low Viscosity resin at 70°C [14]. Subsequently, ultrathin sections (70 nm thick) obtained using the Reichert Ultracut-S ultramicrotome and a glass knife were transferred to redistilled water and stained with a 0.5 M aqueous solution of uranyl acetate and lead citrate [15]. Images were observed and recorded using the FEI Tecnai G2 Spirit Bio TWIN transmission electron microscope at an accelerating voltage of 120 kV. Images were captured using a Megaview G2 Olympus Soft Imaging Solutions camera.

### 2.4. Statistical Analyses

Data for each cultivar was analyzed separately as a standard deviation and the correlation coefficient at the five percent level (the results are shown in Table 1).

## 3. Results

The fruits of “Clapp's Favourite” and “Conference” exhibited inconsiderable morphological differences in the fruit set stage, whereas in the harvest maturity stage they differed distinctly in the colour, shape, and surface. 3-week-old fruit sets in both cultivars had long nonglandular trichomes visible primarily on the calyx. In the harvest maturity stage, broad oval, symmetric “Clapp's Favourite” fruits had a thick, slightly glossy peel with a brown-red fuzzy, point blush, and tiny green lenticels. In turn, the “Conference” fruits were strongly elongated, the peel was matt, rough, and greenish without a blush but with brown russeting over a half of the fruit, and the lenticels had a larger diameter than in the “Clapp's Favourite”.

**Figure 1:**
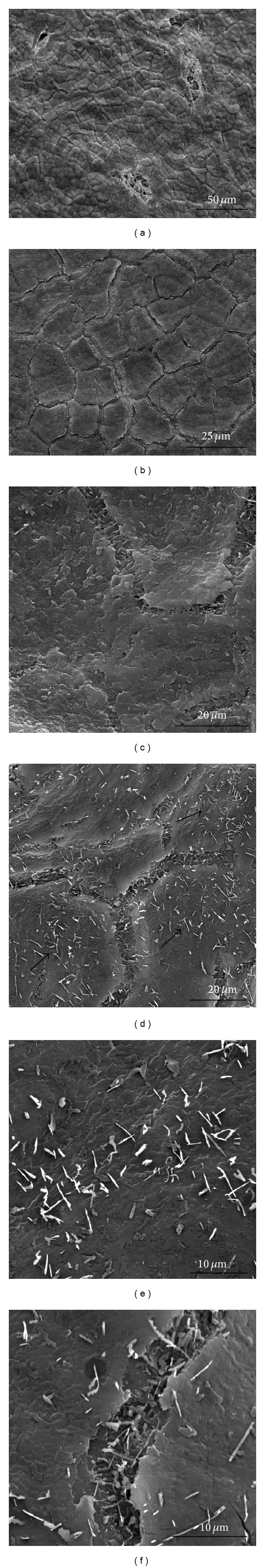
SEM: epidermis surface of the “Conference” ((a), (d)–(f)) and “Clapp's Favourite” ((b), (c)) fruits at the fruit set stage. ((a), (b)) fragments of the epidermis surface with microcracks;((c), (d)) fragments of the epidermis surface with microcracks and horizontal (c) and vertical crystalline wax platelets (arrows) (d); (e) numerous vertical wax platelets between the microcracks; (f) microcracks with few vertical platelets of epicuticular wax.

**Figure 2:**
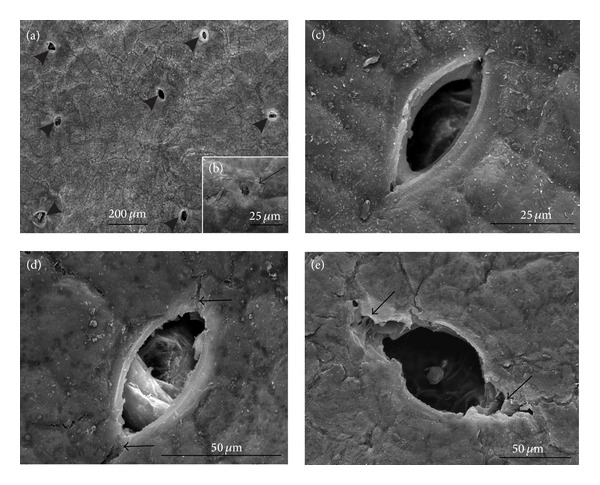
SEM: stomata in the fruit epidermis of the “Clapp's Favourite” ((a), (b), (d)) and “Conference” ((c), (e)) fruits at the fruit set stage. (a) numerous visible stomata (arrowheads); (b) the site (scar) of a broken off mechanical trichome (arrow) is visible; ((c), (e)) open stomata; (d) stoma with structures of unknown origin; ((d), (e)) stoma with epidermis cracks (arrowheads).

*SEM*. The surface of the 3-week-old fruits was slightly undulating. The epidermis was composed of compact cells, whose outer periclinal walls had a tetragonal or pentagonal shape (Figures 1(a) and 1(b)). Besides the surface with a continuous cuticle layer, there were sites of single or more numerous shallow microcracks (Figures 1(a)–1(d)). On the fruit surface in both cultivars, there were poorly visible, merging horizontal platelets of epicuticular wax (Figure 1(c)) and in the “Conference” additional vertical platelets, which were also visible in the microcracks (Figures 1(d)-1(e)). The epidermis of the examined cultivars contained numerous lens-shaped stomata (Table 1 and Figures 2(a) and 2(c)–2(e)) and a few much smaller oval apertures, that is, remainders of lost mechanical trichomes (Figure 2(b)). The stomata were usually open (Figures 2(c) and 2(e)) and sometimes contained structures of unknown origin in the pores (Figure 2(d)). Ruptured epidermis cells, which increased the length of the stomatal pores, were occasionally visible (Figures 2(d) and 2(e)).

**Figure 3:**
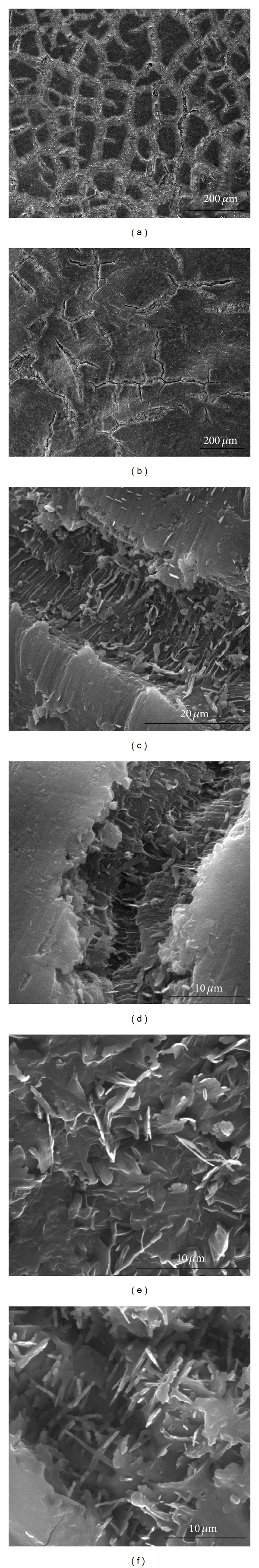
SEM: epidermis surface of the “Clapp's Favourite” ((a), (c), (e), (f)) and “Conference” ((b), (d)) fruits at the harvest maturity stage. ((a), (b)) fragments of the epidermis surface with numerous microcracks; ((c), (d)) microcracks of varying depth; ((e), (f)) vertical and horizontal platelets of epicuticular wax between the microcracks (e) and inside the microcracks (f).

**Figure 4:**
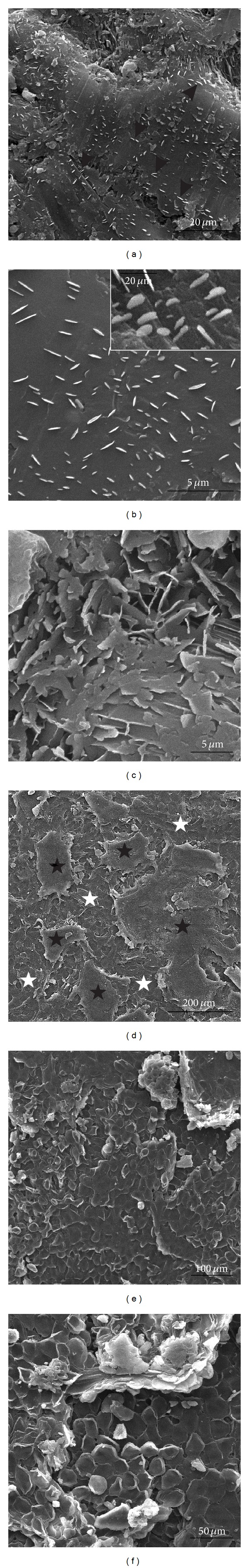
SEM: epidermis surface of the “Conference” fruits at the harvest maturity stage. ((a), (b)) vertical wax platelets on the epidermis surface (arrowheads); (c) vertical and horizontal platelets of the epicuticular wax inside a microcrack; (d) fruit surface with remains of the epidermis (black asterisk) and russeting formed by cork cells (white asterisk); polygonal cork cells were visible within the lenticels (e) and microcracks (f). Note the lack of crystalline wax platelets.

In the harvest maturity stage, the fruit surface in both cultivars was characterised by a great number of microcracks, which formed a specific reticulate network often aligned along the epidermal cell walls (Figures 3(a) and 3(b)). In “Clapp's Favourite,” the microcracks were relatively shallow and superficial (Figure 3(c)), whereas in the “Conference” they reached deeper layers of the cuticle (Figure 3(d)). In the surface of the “Clapp's Favourite” fruits, horizontal and vertical interconnected and merging wax platelets were visible (Figure 3(e)), and a greater number of vertical wax platelets with distinct contours were observed in the microcracks (Figure 3(f)). The surface of the “Conference” fruits was more diverse. Likewise in the “Clapp's Favourite,” at the sites where the fruits were still covered by epidermis there were horizontal and vertical wax platelets; however, their contours were more distinctly outlined (Figures 4(a)–4(c)). The wax platelets in the microcracks were aligned at various angles towards the fruit surface (Figure 4(c)). Additionally, a considerable part of the “Conference” fruit surface was covered by russeting, that is, cork cells, which exfoliated and revealed deeper layers (Figures 4(d)–4(f)). Between the russetings, there were remnants of epidermis in the form of cuticle-covered irregular platforms (Figure 4(d)). No forms of epicuticular wax were found in the lenticels and microcracks present within the russeting.

**Figure 5:**
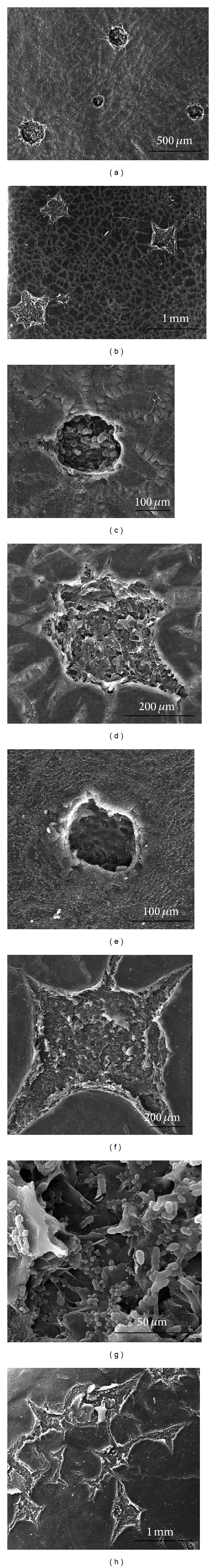
SEM: epidermis surface of the “Clapp's Favourite” ((a), (c), (d)) and “Conference” ((b), (e)–(h)) fruits at the harvest maturity stage with lenticels; ((a), (c), (e)) oval lenticels; ((a), (c)) open lenticels; ((b), (d), (f)) closed star-shaped lenticels; (g) fungal spores and possibly bacterial cells visible inside the lenticel, (h), exfoliating cuticle sheets.

In both cultivars, mature fruits were characterised by a markedly lower number of lenticels per fruit surface unit than in the fruit set stage (Table 1). The lenticels were formed at stomata or at the sites of mechanical trichome loss apertures; their number was 23% lower in the “Conference” than in the “Clapp's Favourite.” The lenticels were oval or star-shaped, varied sizes, and open or closed (Figures 5(a)–5(f)). In “Clapp's Favourite,” the oval lenticels were generally open and filled with loosely arranged spherical cells (Figure 5(c)), whereas the star-shaped lenticels were closed by cuticle, hypodermis cells or polygonal cork cells (Figure 5(d)). In the “Conference,” a majority of the ventilation apertures seemed to be closed (Figures 5(e) and 5(f)). In some lenticels, fungal spores and/or probably bacterial cells were observed (Figure 5(g)). Additionally, exfoliating and detached cuticle patches were observed between the lenticels in the “Conference” fruits (Figure 5(h)).

**Figure 6:**
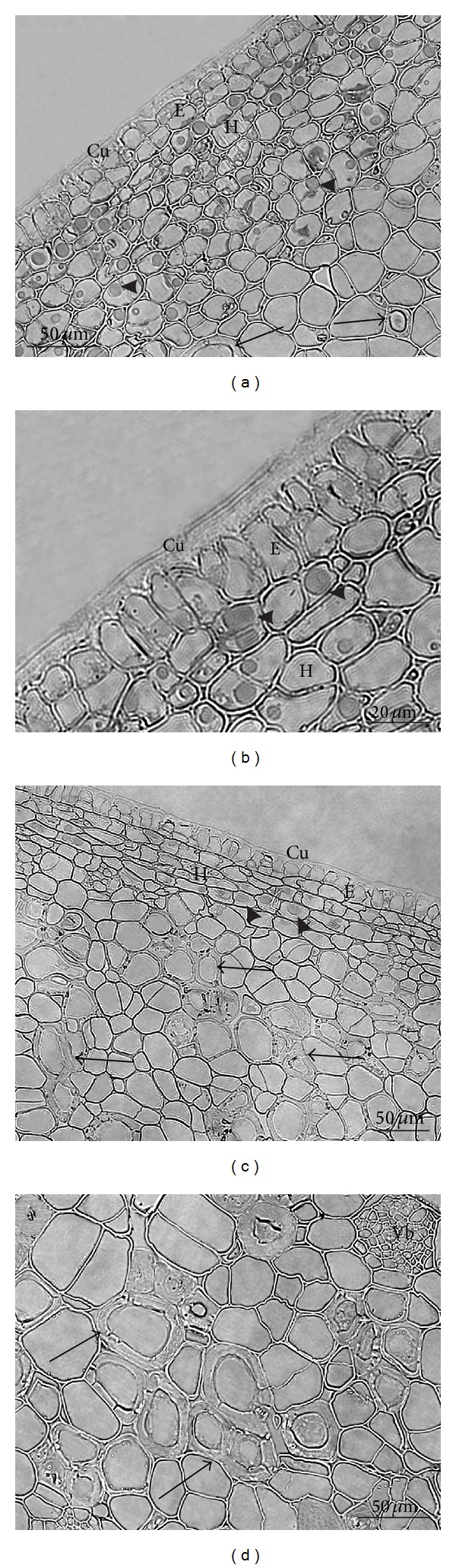
LM: fragments of the cross-sections through the “Clapp's Favourite” ((a), (b)) and “Conference” ((c), (d)) peel at the fruit set stage; ((a)–(c)) visible divisions of the epidermal cells and spherical deposits in the hypodermis (arrowheads); ((a), (c), (d)) stone cells are visible in the parenchyma (arrows); Cu: cuticle, E: epidermis, H: hypodermis, P: parenchyma, and Vb: vascular bundle.

*LM*. Cross sections of the surface layers of 21-day-old fruits of the investigated pear cultivars revealed that the pericarp of the fruit sets was composed of the cuticle (emitting blue light under the fluorescence microscope), epidermis, multilayered hypodermis, and a parenchyma layer (Figures 6(a)–6(d)). In the “Clapp's Favourite,” the cuticle was 32% thicker than the cuticle on the “Conference” fruits (Table 1). The epidermis in both cultivars was formed by rectangular cells, whose height (length of anticlinal walls) was greater than the width (length of periclinal walls) (Figures 6(a)–6(c)). In this stage of fruit development, the epidermis exhibited features of meristematic tissue, as divisions of its cells were noted. At some sites, the epidermis was single-layered and divisions were visible along anticlinal walls; at other sites, the epidermis formed two cell layers as a result of division along the periclinal walls (Figures 6(a)–6(c)). The height of epidermis cells in the “Clapp's Favourite” was 18% greater than the height of epidermis cells in the “Conference” (Table 1). The hypodermis was formed by tiny, longitudinally, and laterally dividing cells with relatively thin walls (Figures 6(a)–6(c)). The hypodermis layer in “Clapp's Favourite” was 20% thicker than in the “Conference” (Table 1). The hypodermis cells contained numerous chloroplasts emitting red autofluorescence and different-sized brown spherical deposits that were particularly visible in the “Clapp's Favourite” and were not stained by FeCl_3_ (Figures 6(a) and 6(b)). In the parenchyma, there were dividing cells and, additionally, inclusions of stone cells emitting blue light under the UV filter (Figures 6(a), 6(c), and 6(d)).

**Figure 7:**
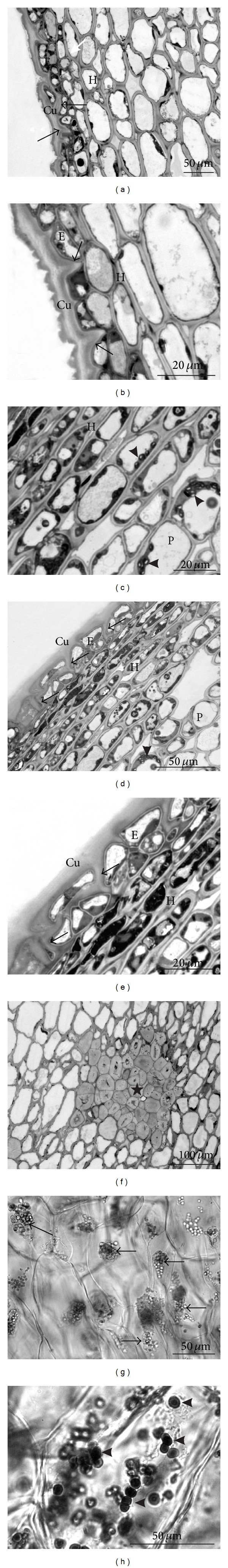
LM: fragments of the cross-sections through the “Clapp's Favourite” ((a), (b)) and “Conference” ((c)–(h)) fruit peel at the harvest maturity stage; ((a)–(d)) visible cuticle penetrating the anticlinal and internal periclinal walls of the epidermis (arrows) and an irregular shape of the epidermis cells; ((c)–(e)) visible dark deposits of tannin compounds in the epidermis and hypodermis cells and plastids with starch grains in the hypodermis and parenchyma (arrowheads) (c); (f) stone cell aggregation in the parenchyma (asterisk); (g) leucoplasts with starch grains in the parenchyma (staining with IKI) (arrows); (h) deposits of tannin compounds in the parenchyma (staining with FeCl_3_) (arrowheads); Cu: cuticle, E: epidermis, H: hypodermis, and P: parenchyma.

In the stage of harvest maturity, the thickness of the cuticle increased by 24% in the “Clapp's Favourite” and 26% in the “Conference” in comparison with the fruit set stage. However, like in the fruit set stage, the cuticle in the “Clapp's Favourite” fruits was 23% thicker (Table 1). In the external cuticle layers in both cultivars, numerous sites exhibited cracks and penetration of the cuticle into anticlinal and inner periclinal epidermis walls, which increased the thickness of this tissue (Figures 7(a)–7(d)). In turn, the height of epidermis cells in both cultivars was reduced by over 40% compared with that in the fruit set stage, and the cells had highly irregular shapes (Figures 7(a)–7(d)). Moreover, the epidermis on the “Clapp's Favourite” fruits was still 19% thicker in comparison with that in the “Conference” fruits (Table 1).

Hypodermis cells formed of patches of collenchyma with thickened tangential walls were elongated along the periclinal walls, likewise the epidermis cells (Figures 7(a)–7(e)). In the “Conference” fruits, they were smaller and flatter (Figures 7(c)–7(e)). The thickness of this tissue layer was 28% lower in the “Clapp's Favourite” and 41% lower in the “Conference” in comparison with the hypodermis thickness in the fruit set stage (Table 1). Nevertheless, the thickness of the cuticle layer was still 31% greater in the “Clapp's Favourite.” In the hypodermis vacuoles, there were numerous, different-sized, spherical deposits of tannin compounds (Figures 7(a), 7(c), and 7(d)) staining dark brown-purple with FeCl_3_. Additionally, numerous plastids containing starch grains were visible in the cytoplasm of the hypodermis cells in the “Conference.”

In both cultivars, deposits of cuboid and cube calcium oxalate crystals and aggregates of stone cells were visible in the innermost hypodermis layers and the outer parenchyma layers. In the fruit set stage in the “Clapp's Favourite” fruits, sclereids aggregates formed 2-3-cell clusters or were single, whereas in the “Conference” they were more numerous and contained several cells (Figures 6(a), 6(c), and 6(d)). Sclereid aggregates composed of several cells were found in harvest-mature fruits of both cultivars, with a greater number thereof in the “Conference” (Figure 7(f)). The largest number of sclereids in both cultivars was located in the inner, ca. 5 mm thick pericarp layer; additionally, in the “Clapp's Favourite,” they were present in a close vicinity of the receptacle, and in the “Conference” around the area of the fruit calyx. The diameter of stone cells and the thickness of their walls in both cultivars were similar (Table 1).

During fruit development, the size of parenchymal cells also increased in both cultivars by 3% in “Clapp's Favourite” and over 19% in “Conference.” In the fruit set stage, the thickness of the layer extending over three parenchyma layers was 12% higher in the “Clapp's Favourite”; in turn, the thickness of the parenchyma layer in the harvest maturity stage was by ca. 6% higher in the “Conference” fruits (Table 1). Furthermore, only the “Conference” exhibited numerous chloroplasts containing starch grains (Figure 7(g)) and tiny, equally numerous spherical deposits of tannin compounds in the cells of deeper parenchyma layers (Figure 7(h)).

**Figure 8:**
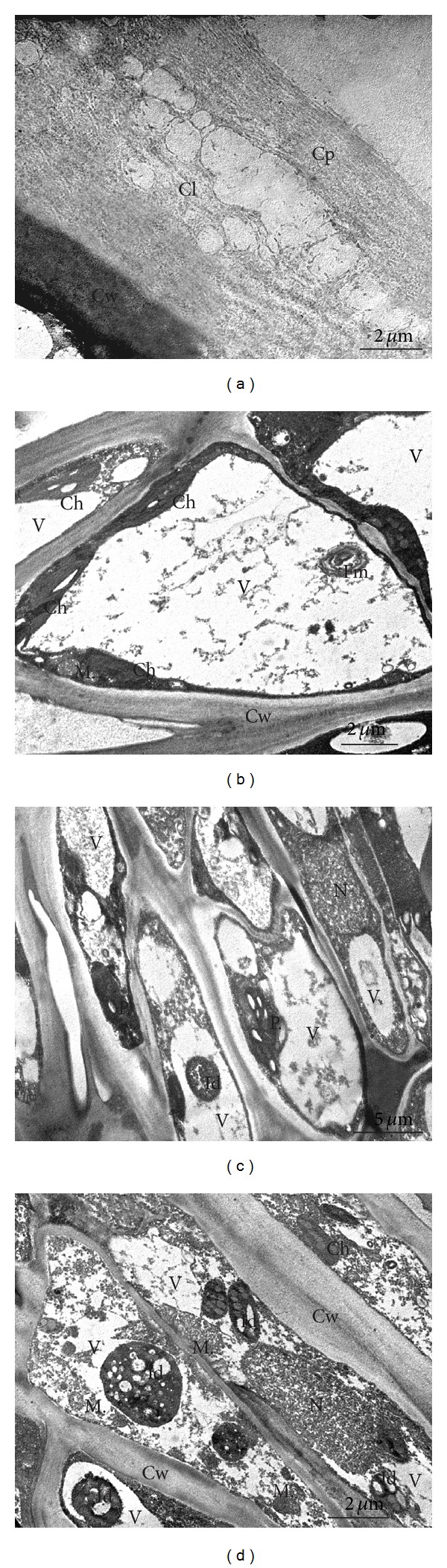
TEM: ultrastructure of the “Conference” ((a)–(c)) and “Clapp's Favourite” (d) fruit peel at the fruit set stages; (a) fragment of the fruit cuticle; (b) epidermis cell; ((c), (d)) hypodermis; Cp: cuticle proper, Cl: cuticular layer, N: nucleus, Ch: chloroplasts with starch grains, M: mitochondrion, V: vacuoles, P: plastids with starch grains, Mf: myelin figure, Id: intravacuolar deposits, and Cw: cell walls.

*TEM*. No distinct differences between the investigated cultivars were found in the ultrastructure of cells of fruit peel and parenchyma. Two layers, that is, cuticle proper and a cuticular layer, were observed in the cuticle of fruit sets in both cultivars (Figure 8(a)). In the “Conference,” the cuticular layer was composed of a reticulate layer adjacent to the cell wall and an internal layer with visible numerous different-sized vesicles and empty spaces (Figure 8(a)). In turn, the outer cuticle layer forming the cuticle proper had a lamellate structure (Figure 8(a)). The cytoplasm of the epidermis cells contained cell nuclei, mitochondria, chloroplasts containing a few starch grains, and endoplasmic reticulum (Figure 8(b)). The hypodermis in the fruits of both cultivars exhibited chloroplasts and numerous different-sized and -shaped plastids with starch grains (Figures 8(c) and 8(d)). The epidermis and hypodermis vacuoles contained dark deposits, fibrous residues, and myelin figures (Figures 8(b)–8(d)).

**Figure 9:**
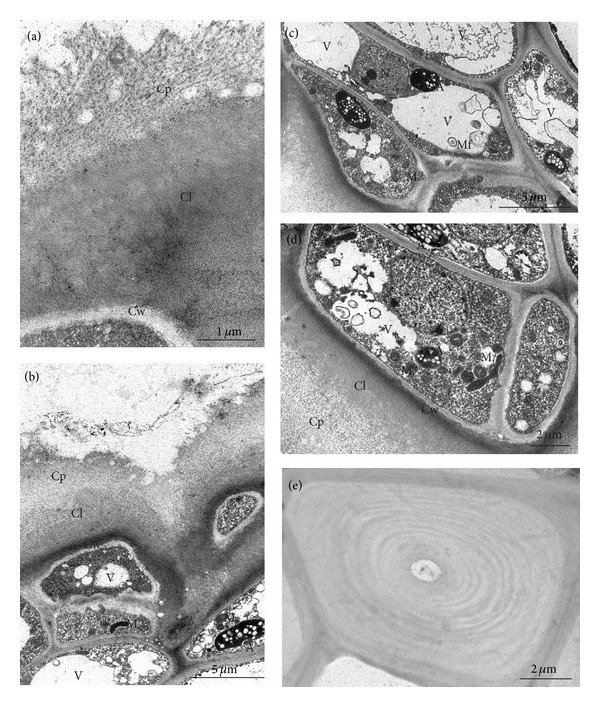
TEM: ultrastructure of the “Conference” fruit peel at the harvest maturity stage; (a) fragment of the fruit cuticle; (b) fragment of epidermis with cuticle and hypodermis; (c) epidermis cells with myelin figures and fibrous residue in the vacuoles; (d) epidermis and hypodermis cells; (e) stone cells in the hypodermis; Cp: cuticle proper, Cl: cuticular layer, A: amyloplasts with starch grains, M: mitochondrion, Mf: myelin figures, V: vacuoles, and Cw: cell walls.

In the harvest maturity stage, the fruit cuticle had a reticulate structure; however, the cuticle proper was characterised by an irregular surface and a less compact structure (Figures 9(a) and 9(b)). The cytoplasm of epidermis and hypodermis cells contained cell nuclei, leucoplasts with small starch grains (exclusively in the “Conference”), numerous mitochondria, and ER (Figures 9(b)–9(d)). In the vacuoles of these cells, there were myelin figures and, probably, fragments of membranes (Figures 9(c) and 9(d)). Moreover, the hypodermis exhibited inclusions of stone cells with narrow lumen and distinct lignin lamellae deposited via apposition (Figure 9(e)).

## 4. Discussion

Fruit quality both after harvest, on the shop shelf, and after storage is important for pear consumers; it is determined by such indicators as flavour, aroma, colour, type of the surface, and firmness. “Clapp's Favourite” and “Conference” fruits differ in the time of harvest and consumption maturity, type of the surface, length of shelf life, and storability. Tao et al. [16] showed in other pear cultivars that the aforementioned traits are genetically conditioned and are associated with the structure of the fruit at the tissue and cellular level.

Many reports concerning the causes of decreased fruit firmness emphasise the role of the peel, that is, the surface layer composed of the cuticle-covered epidermis and several layers of the hypodermis. This layer has a pivotal importance for protection of the fruit interior against adverse bio- and abiotic factors and loss of water, which enhances the possibility of prolonged storage of fruits [17–19]. During fruit development, the author of the present paper observed an increase in cuticle thickness in both cultivars; however, the cuticle was thicker in the “Clapp's Favourite” fruits, that is, the nonrusseted cultivar. According to Jackson [12], Tao et al. [16], and Bain [20] the thick cuticle present on nonrusseted pear fruits promotes long-term storage. Furthermore, Amarante et al. [21, 22] and Veraverbeke et al. [23, 24] report that the thicker the cuticle is, the more efficient in preventing fruit transpiration it is. However, in the case of nonrusseted “Clapp's Favourite” fruits, a thicker cuticle does not guarantee either a prolonged storage period or retaining fruit firmness.

Many authors report that the number and type of lenticels per unit area of the epidermis have an impact on the intensity of fruit transpiration [25–28]. It was observed in the present study that the number of lenticels in the harvest maturity stage was similar in both investigated cultivars. However, a majority of lenticels in the “Conference” were closed and only a few were open, whereas many lenticels in the “Clapp's Favourite” were open, which may have caused a higher transpiration rate. According to Maguire et al. [26] and Veraverbeke et al. [27], water transpiration can only proceed via open lenticels.

In the cuticle of the “Clapp's Favourite” and “Conference” fruits, the same number of microcracks was found in both cultivars; however, in the “Conference,” they were deeper and often reached inner cuticle layers. Many researchers believe that microcracks are the so-called “weak points” on the fruit surface that enhance transpiration [24, 26, 27, 29]. However, inside the microcracks in the “Conference” there were numerous vertical crystalline wax platelets, which effectively sealed the microcracks, thereby reducing their permeability. According to many researchers, the presence of wax coating on the fruit surface, and vertical wax platelets in particular, guarantees retaining proper fruit firmness and long-term storability [24, 25, 28–31].

Another contributory factor in protection of the fruit interior against loss of moisture is russeting visible over a large surface area in the “Conference” fruits. Russetings are sites on the fruit surface that are covered by cork cells instead of epidermis. They are associated with the activity of phellogen deposited in subepidermal cells typically at the microcrack formation site. Khanal et al. [32] report that due to its limited permeability and relatively high plasticity, the cork tissue protects the fruit interior against water loss, infections, adverse climatic conditions, and physical factors more efficiently than the epidermis with the cuticle. Currently, pear russeting is regarded as a desirable trait, although it was previously believed to reduce the quality and the market value of the fruit [5, 33, 34].

During the development of the “Clapp's Favourite” and “Conference” fruits, the size of the stone cell aggregates was observed to increase. The stone cells in the “Conference” were more numerous and formed more abundant clusters than in the “Clapp's Favourite,” but their diameter and the thickness of cell walls were similar. Similar observations concerning the development of stone cells in fruits of other cultivars were presented by Bain [20], Nie et al. [35], and Tao et al. [36]. Many authors regard the number and size of stone cells as a genetically conditioned variety-specific feature [37–40]. Furthermore, Liu et al. [41] and Tian et al. [42] suggest that the presence, number, and location of sclereids largely determine fruit hardness. According to Tao et al. [36, 43], the number of sclereids in pear parenchyma determines long-term storability of fruits and the length of their shelf life. Cultivars with stone cells (with lignified cells in the skin) lose lower amounts of water through microcracks and lenticels than these with nonlignified cells [22].

The author of the present study observed numerous intensively FeCl_3_-staining deposits of tannin compounds in all hypodermal and parenchymal layers in the “Conference” fruits. Their amount in the cells of the “Clapp's Favourite” fruits was substantially lower and limited only to the hypodermis. Accumulation of tannin compounds in fruits of various pear and apple cultivars has been reported by other researchers as well [16, 19, 44]. Lees et al. [44] have found that a high content of tannin compounds, which have preservative-bactericidal activity, enhances fruit storability. In the present paper it has been observed that high amounts of tannin materials are frequently accompanied by great numbers of stone cells.

Sugars were accumulated during the development and ripening of the fruits of the examined pear cultivars. Initially, these were polysaccharides in the form of starch grains, which were hydrolysed in the harvest maturity stage in the “Clapp's Favourite” and remained in the solid form for a long time in the “Conference” fruits. According to Tharanathan [45], the presence of semicrystalline starch contributes to the maintenance of proper fruit texture. Concurrently, during the ripening period, there is an increase in the activity of enzymes responsible not only for disintegration of carbohydrates but also for degradation of the cell wall (enzymatic degradation and dissolution of protopectins) and increased ethylene production. Many authors report that the most important processes that impair pear quality are related to turgor loss and degradation of parenchymal cell walls, dissolution of the middle lamella, and gradual disintegration of fibrillar material throughout the cell wall [46–49].

## Figures and Tables

**Table 1:** Characteristics of “Clapp's Favourite” and “Conference” fruits.

Parameters [*µ*m]	Fruit set stage (May)		Harvest maturity stage (September)	
	Clapp's Favourite	Conference	Clapp's Favourite	Conference
Number of stomata (May) and lenticels (September) [in mm^2^]	700 ± 245a	900 ± 316b	26 ± 7a	24 ± 9b
Thickness of cuticle	9.28 ± 0.62	7.03 ± 0.83	11.48 ± 1.06	8.86 ± 0.94
Hight of epidermis cells	23.18 ± 1.4c	19.03 ± 1.6d	13.44 ± 0.93c	10.9 ± 1.37d
Thickness of hypodermis	78.64 ± 7.56e	65.77 ± 6.22f	61.32 ± 7.58eg	46.75 ± 7.32fg
Number of hypodermis layers	5 ± 2	4 ± 2	5 ± 2	6 ± 2
Diameter of stone cells	38.5 ± 2.5	38.1 ± 1.8	37.8 ± 2.2	38.2 ± 2.1
Thickness of the three parenchyma layers	73.05 ± 6.36	66.75 ± 3.72	75.24 ± 8.27	79.69 ± 3.1

a, b, c, d, e, f, g: indicate pairs of traits that differ significantly at *P* < 0.05.
